# Impact of COVID-19 on routine childhood immunisations in low- and middle-income countries: A scoping review

**DOI:** 10.1371/journal.pgph.0002268

**Published:** 2023-08-23

**Authors:** Milena Dalton, Benjamin Sanderson, Leanne J. Robinson, Caroline S. E. Homer, William Pomat, Margie Danchin, Stefanie Vaccher

**Affiliations:** 1 Burnet Institute, Melbourne, Victoria, Australia; 2 Population Health & Immunity Division, Walter and Eliza Hall Institute of Medical Research, Parkville, Victoria, Australia; 3 Department of Medical Biology, University of Melbourne, Melbourne, Australia; 4 Papua New Guinea Institute of Medical Research, Madang, Papua New Guinea; 5 Papua New Guinea Institute of Medical Research, Goroka, Papua New Guinea; 6 Department of General Medicine, The Royal Children’s Hospital, Parkville, Victoria, Australia; 7 Vaccine Uptake Group, Murdoch Children’s Research Institute, Parkville, Victoria, Australia; 8 Department of Paediatrics, University of Melbourne, Melbourne, Victoria, Australia; 9 Burnet Institute, Port Moresby, Papua New Guinea; Public Health Foundation of India, INDIA

## Abstract

Routine vaccines are critical to child health. The COVID-19 pandemic significantly impacted essential health services, particularly in low-and middle-income countries (LMICs). We reviewed literature to determine the impact of COVID-19 on service delivery and uptake of routine childhood immunisation in LMICs. We reviewed papers published between March 2020 and June 2022 using a scoping review framework, and assessed each paper across the World Health Organisation health system strengthening framework. Our search identified 3,471 publications; 58 studies were included. One-quarter of studies showed routine childhood immunisation coverage declined (10% to 38%) between 2019 to 2021. Declines in the number of vaccine doses administered (25% to 51%), timeliness (6.2% to 34%), and the availability of fixed and outreach services were also reported. Strategies proposed to improve coverage included catch-up activities, strengthening supply chain and outreach services. Re-focusing efforts on increasing coverage is critical to improve child health and reduce the likelihood of disease outbreaks.

## 1. Introduction

Immunisation is a life-saving and cost-effective public health intervention [1]. Routine childhood immunisation is an essential component of child healthcare globally [2]. The Expanded Programme on Immunisation (EPI) was established in 1974 to strengthen routine immunisation programs across all World Health Organisation (WHO) regions [3]. Routine vaccines included in the EPI include the Bacillus Calmette–Guérin (BCG) vaccine, Hepatitis B (Hep B) vaccine, polio, Diphtheria, Tetanus, Pertussis (DTP) containing vaccine, Haemophilus influenzae type b (Hib) vaccine, Pneumococcal conjugate vaccine (PCV), Rotavirus vaccine (Rota), Measles and Rubella [4]. WHO regions are divided into the African Region, Region of the Americas, South-East Asia Region, European Region, Eastern Mediterranean Region, and the Western Pacific Region.

WHO declared COVID-19 as a global pandemic on 11 March 2020 [5]. Since then, the COVID-19 pandemic has disproportionately disrupted already fragile health systems and health services, such as routine childhood immunisation. UNICEF estimates that there were 67 million children who missed out on all or some routine vaccines between 2019 and 2021 [6]. Of these 67 million UNICEF estimates that 48 million received no routine vaccines during this period [6]. This has set back routine immunised coverage to levels prior to 2008 [6]. Of these children, 60% live in 10 low- and middle-income countries (LMIC) including Angola, Brazil, the Democratic Republic of the Congo (DRC), Ethiopia, India, Indonesia, Myanmar, Nigeria, Pakistan, and the Philippines [7].

LMICs comprise 63% of countries globally. To address the backslide that the COVID-19 pandemic has caused for routine childhood immunisation services in LMICs, we need to understand what impact the pandemic has had in LMICs more broadly [8]. This needs to be done within a health system strengthening framework to identify strategies that could be used to strengthen multiple components of the health system. WHO describes health systems in terms of six building blocks [9]. We have applied this health system strengthening lens to our analysis, identifying which of the six WHO health systems strengthening building blocks each study focuses on.

This review aimed to systematically examine the literature to determine the impact of COVID-19 on routine childhood immunisation in LMICs, identify research gaps and summarise research findings. This review also highlights strategies that could be used to repair and strengthen routine immunisation services moving forward within the context of the health system.

## 2. Methods

### 2.1. Search strategy

A comprehensive search strategy was developed in consultation with a medical librarian (S2 Table). The team followed PRISMA guidelines (S1 PRISMA Checklist) and registered the review at Open Science Framework. Keywords were developed from a number of thesauri including Medical Subject Headings (MeSH) databases for appropriate headings and text words [10]. The search strategy and key search terms are outlined in Appendix 1. Key terms included “COVID-19” and “routine immunisation” and “low- and middle-income countries”. This income classification, LMIC, has been assigned to countries by the World Bank and is based on a country’s Gross National Income [11].

The search strategy was applied to three databases: MEDLINE (OVID), EMBASE (OVID) and Global Health (OVID). It was also applied to Medrxiv to capture any relevant pre-print articles. Google scholar was also searched using a key terms search strategy. A comprehensive search of grey literature, which included Google Scholar, the WHO website, the United Nations Children’s Fund website and the Gavi website was also conducted [2,12,13]. A manual search of reference lists of key included articles was conducted to ensure that all relevant articles were captured in the search. The search was conducted from June 7 to 10, 2022.

### 2.2. Inclusion and exclusion criteria

**Fig 1:**
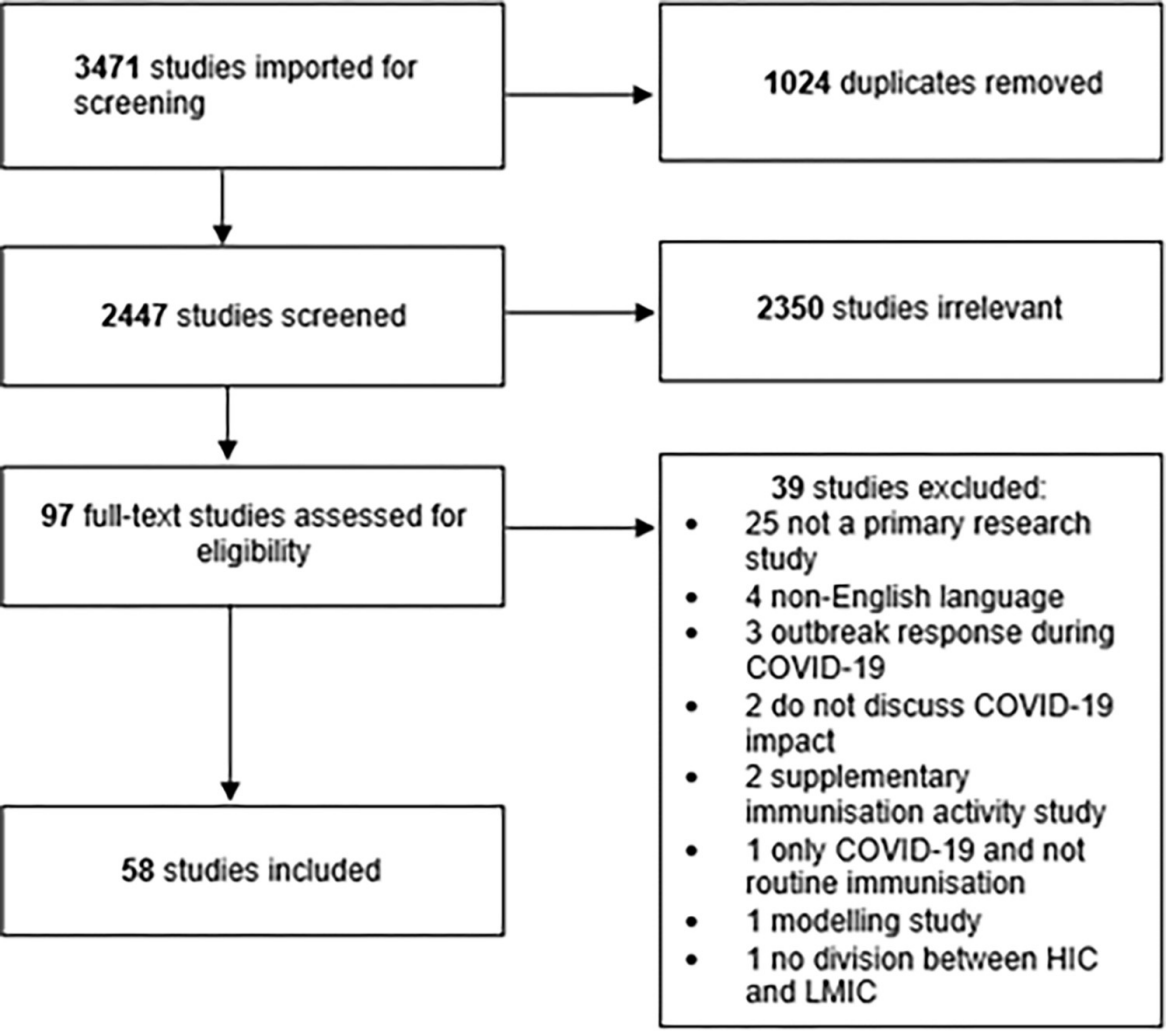
PRISMA flow diagram.

The inclusion criteria were only original research studies which included information on the impact of COVID-19 on routine childhood immunisation and studies which focused on LMICs. Fig 1 displays the PRISMA flow diagram, which demonstrates the review of 97 studies resulting in 58 studies included in the qualitative synthesis. Exclusion criteria included non-English language and published before 2020 (Fig 1).

### 2.3. Data processing

All citations were imported into Covidence. Duplicates were removed and two reviewers (MD and BS) conducted initial screening of the remaining studies and then a full-test review of eligible studies. Discrepancies were resolved through discussion.

### 2.4. Data extraction and quality assessment

We developed a comprehensive data extraction form using Covidence [14]. Reviewers (MD and BS) tested this form with five articles. Recorded study features included authors, year of publication, country, LMIC status and WHO region, and the data extraction form collected specific data points related to routine childhood immunisation and health systems strengthening [15]. For measles and polio, where information on the specific vaccine was not available, we classified the vaccine as ‘measles containing’ or ‘polio containing’ vaccine, respectively. Additionally, reviewers noted the study design and sample size for each article. No studies were excluded based on these characteristics.

### 2.5. Data analysis

We undertook a descriptive numerical summary analysis of the extracted data to ascertain major findings, study characteristics including publication year, study type, countries and regions in which the studies were conducted, antigens studied and service delivery characteristics which included the study setting, health system level, facility ownership and service type [16]. We conducted a thematic analysis to ascertain additional detail on routine childhood immunisation coverage [16]. This included doses administered, fixed and outreach services provided, delayed or missed vaccination, vaccination timeliness, the most common factors contributing to reduced immunisation coverage, the most commonly proposed strategies to improve routine childhood immunisation service delivery and which health system strengthening categories each study focused on.

For the health system strengthening analysis we used the WHO health system strengthening framework to guide global health system strengthening efforts [9]. This framework outlines six building blocks for a health system: i) service delivery, ii) health workforce, iii) health information system, iv) medical products, vaccines and technologies, v) financing and vi) leadership and governance. With these building blocks in place there should be an increase in access, coverage, quality and safety leading to improved health, responsiveness of the system, social and financial risk protection and improved efficiency. In 2010, WHO published *Monitoring the building blocks of health systems*: *A Handbook of Indicators* and strategies to measure each building block to help track progress in strengthening health systems. In this handbook, building block iv, medical products, vaccines and technologies, was reclassified as access to essential medicines [17].

When considering which of the building blocks were included in each study, we used the following categories, as per the WHO’s health systems strengthening framework: [9]

- **Service delivery:** packages, delivery models, infrastructure, management, safety and quality and demand for care.
- **Health workforce:** national workforce policies and investment plans, advocacy, norms and standards and data.
- **Health information systems:** facility and population-based information and surveillance systems, global standards and tools.
- **Access to essential medicines:** norms, standards, policies, reliable procurement, equitable access and quality.
- **Financing:** national health financing policies, tools and data on health expenditures and costing.
- **Leadership and governance:** health sector policies, harmonization and alignment, and oversight and regulation.

## 3. Results

### 3.1. Study characteristics

Fifty-eight studies met the criteria for inclusion and data extraction (Fig 1). Most studies (59%) were published in 2021 and used cross-sectional methods (Table 1). However, it is important to note that most data were collected in 2019 and 2020, before the COVID-19 vaccine roll-out began. Data on 13 different vaccines was included (Table 2). The polio vaccine was the most studied routine childhood vaccine. Studies were conducted in five WHO regions with the majority conducted in lower-middle income countries in these regions.

**Table 1:** Characteristics of included studies (N = 58).

		No. of studies reporting N(%)
**Year of publication**		
	2020	4 (7)
	2021	34 (59)
	2022	20 (34)
**Study Type**		
	Cross-sectional	43 (74)
	Ecological	6 (10)
	Mixed methods	5 (9)
	Qualitative	3 (5)
	Retrospective cohort	1 (2)
*WHO Region		
	African	23 (40)
	Region of the Americas	14 (24)
	Western Pacific	12 (21)
	Eastern Mediterranean	9 (16)
	South-East Asia	3 (10)

**Table 2:** Antigen type.

Antigen (N = 58)		No. of studies reporting *N(%)
	BCG	36 (62)
	Pentavalent vaccine (DTP, HiB, Hepatitis B)	35 (60)
	PCV	25 (43)
	Rotavirus	22 (38)
	Hepatitis B	11 (19)
	DTP	10 (17)
	Meningococcal C	4 (7)
	Yellow fever	3 (5)
	Hepatitis A	3 (5)
	Varicella	2 (3)
	Tetravalent vaccine (DTP, HiB)	1 (2)
	Hib	1 (2)
	Japanese encephalitis	1 (2)
**Polio-containing vaccine (N = 47)**		
	OPV	25 (53)
	IPV	19 (40)
	Polio vaccine (not otherwise specified)	3 (6)
**Measles-containing vaccine (MCV) (N = 40)**		
	(M)MR	20 (50)
	MCV (not otherwise specified)	20 (50)

BCG = Bacillus Calmette–Guérin; DTP = Diphtheria, Tetanus, and Pertussis; Hib = Haemophilus influenzae type b; PCV = Pneumococcal Conjugate Vaccine; OPV = Oral Polio Vaccine; IPV = Inactivated Polio Vaccine; MCV = Measles-Containing Vaccine; Measles Mumps and Rubella = (M)MR

*Note: Some papers reported on COVID-19 impacts in multiple countries, regions, or antigens, so percentages may be greater than the total number of papers included (N = 58).

### 3.2. Service delivery characteristics

Most of the papers which stated the study setting were conducted in urban areas (Table 3). Sixteen of the studies were conducted in rural areas and six in peri-urban areas. Data were collected slightly more at the tertiary hospital level compared to the community, primary and secondary level and in publicly funded facilities. Almost two-thirds of studies included fixed site services and one-fifth included outreach services.

**Table 3:** Service delivery characteristics.

		No. of studies reporting N(%)*
Study Setting		
	Urban	21 (36)
	Peri-Urban	6 (10)
	Rural	16 (28)
	Urban and Rural	10 (17)
	Urban, Peri-Urban and Rural	2 (3)
	Not stated	31 (53)
Level in the health system		
	Community	14 (24)
	Primary	17 (29)
	Secondary	15 (26)
	Tertiary	20 (34)
	Not stated	10 (17)
Facility ownership		
	Public	24 (41)
	Private	14 (24)
	Not stated	33 (57)
Service type		
	Fixed	37 (64)
	Outreach	12 (21)
	Not stated	21 (36)

*Note: Some papers reported on COVID-19 impacts in multiple study settings, health system level and service type, so percentages may be greater than the total number of papers included (N = 58).

### 3.3. Overview of COVID-19 impact on routine childhood immunisations

A variety of studies were identified (S1 Table). Fifty-two were single-country studies, with only 10% being multi-country studies. Most studies focused on the impact of COVID-19 on immunisation coverage and service delivery. This included the impact on vaccination timeliness and the change in the number of doses administered. Eight studies used a mixed-methods or qualitative approach to investigate more contextual factors impacting routine immunisation service delivery during the pandemic [18–25]. Three studies also examined caregiver attitudes towards routine childhood vaccines [24,26,27]. Many of the studies provided a comparative analysis using data from before the COVID-19 pandemic with data from during the pandemic to demonstrate the change. Eight studies presented findings on the impact of COVID-19 on routine immunisation within a broader study on the impact on reproductive, maternal or child health services [28–35].

### 3.4. Coverage declines

One quarter of included studies reported a decline in routine childhood immunisation coverage [21,32,34,36–47]. Approximately 80% of these studies were conducted in either the African region or the Region of the Americas. The overall decline in coverage for all routine vaccines from 2019 to 2020 ranged from 10% to 38% [21,39]. Reported decline in MCV ranged from 10% to 48% [45,47]. For Pentavalent3, this ranged from 4.1% to 37% and BCG from 20% to 47% [21,37,45,47].

### 3.5. Decline in number of vaccine doses administered

One fifth of studies reported a decline in vaccine doses administered [18,28,37,48–57]. Overall declines in vaccine doses administered ranged from approximately 25% to 51% [56,58]. Hepatitis B, BCG, MCV and DTP-containing vaccine were most commonly discussed. For example, Babatunde et al. found that the administration of post-natal antigen BCG fell by 3.7% and Hepatitis B by 3.5% in Oyo State of Nigeria [37]. For the third dose of Pentavalent, the cumulative reduction in doses administered between March and July 2020 ranged from 2% in Cameroon to 17% in Mali [28].

### 3.6. Decline in fixed and outreach services

Several studies reported on the reduction of fixed services, outreach services and service utilisation. Four studies reported a decline in fixed services provided during the study period [18,37,38,59]. Another four studies also reported a decline in or limited outreach services provided [18,37,38,58]. For example, Babalola et al. found that fixed services were offered by 92% of facilities included in the Liberian study, but there was a 47% decline in outreach services [18]. Similarly, Babatunde et al. reported that no clinic completed all of their planned fixed or outreach sessions [37]. A study by Hanifi et al. reported that 71% of outreach sessions in Bangladesh were suspended between March and May 2020 [22]. Four studies reported a decrease in routine immunisation service utilisation [19,35,60,61].

### 3.7. Delayed or missed vaccination

One quarter of studies reported delayed or missed vaccination [24,25,41,46,62–70]. Overall delayed vaccination ranged from 6.2% to 34% [62,64]. The delay for Hepatitis B ranged from 5% to 6.3% [65,70]. For BCG, this delay ranged from 3.7% to 55.4% [65,70]. Jain et al. however, reported no difference in the timeliness of measles vaccinations after lockdowns compared to before lockdowns [41]. For studies reporting on missed vaccination, Murthy et al. reported that 34% of children included in the study missed at least one scheduled vaccine while Tegene et al. found that 62% of children that participated in the study did not complete their schedule in the South Region of Ethiopia [25,67]. Tegene et al. also found that 14.6% of children aged 10 to 23 months had never received a vaccine. This is the only study that explicitly identified children who had never been vaccinated. Powelson et al. found that partially vaccinated children in Zambezia province, Mozambique were missing 5.1 vaccines on average by the time they were two years of age [24].

### 3.8. Limited impact on routine immunisation

Despite reported declines in several components of health service delivery, several studies reported no significant impact on some aspects of routine immunisation. For example, Alves et al. reported no significant decrease in the number of immunisation doses per child between 2017 and 2020 and no significant impact of COVID-19 isolation measures on the number of vaccines per child after March 2020 in Brazil [71]. In a similar way, Bekele et al. reported that routine immunisation remained stable during the first six months of the COVID-19 pandemic in the North Shewa zone of Ethiopia [20]. Again, Connolly et al. found that none of their country sites (Haiti, Lesotho, Liberia and Malawi) had a statistically significant decline in the percentage of vaccines administered at birth in the period from March to August 2020 [72]. The Bello et al. study of 19 countries in East and Southern Africa, found that Kenya, Mozambique and Tanzania had an increased proportion of fixed and outreach sessions as well as an increase in DTP2 and MCV1 coverage when comparing January to August 2020 to January to August 2019 [38].

In a study conducted in Kinshasa, the capital of the DRC, Hategeka et al. found that overall COVID-19 did not significantly affect vaccinations administered [73]. Another study that included data from DRC supported these conclusions [28]. Shapira et al. found that for seven of the eight countries included in the study, Cameroon, Liberia, Malawi, Mali, Nigeria, Sierra Leone and Somalia, the number of children who received the third dose of Pentavalent vaccine dropped for at least one month [28]. There was no drop in children who received the third dose of Pentavalent vaccine in the eighth country, the DRC. Kinikar et al. found that there was some decline in the number of newborn immunisations at a tertiary teaching hospital in India but that this was not statistically significant when compared to pre-COVID-19 lockdown figures [51]. In Kenya, Shikuku et al. found that there was no significant change in the mean total immunisation service hospital attendance per month from March to June 2020 compared with the equivalent four-month period in 2019 [30]. Wambua et al. also found that overall immunisation services remained unaffected in Kenya [31].

### 3.9. Factors impacting routine childhood immunisation

Ninety percent of studies discussed specific factors that contributed to changes in routine childhood immunisation coverage during the COVID-19 pandemic. Many of these factors negatively impacted coverage, with the most common factors highlighted in Table 4 below.

**Table 4:** Most common factors identified from reviewed articles contributing to a reduction in immunisation coverage.

1. Fear of being exposed to COVID-19 2. Lockdown movement restrictions 3. Reduced healthcare services 4. Public transport disruption 5. Supply chain disruption 6. Poor government or media messaging 7. Human resource redeploymen 8. Healthcare worker availability

Four studies also highlighted factors that had a positive impact on routine childhood immunisation coverage [20,30,31,38]. This included a ministry of health prioritising routine immunisation, particularly measles, during the COVID-19 pandemic and the deployment of health extension workers to health posts for community-based services, including routine immunisation. A well-coordinated and centralised government risk communication strategy which ensured critical essential health services continued and designated health facilities for vaccination to offset vaccination centres assigned as COVID-19 isolation centres were also highlighted as factors which had a positive impact.

### 3.10. Health system strengthening

**Fig 2:**
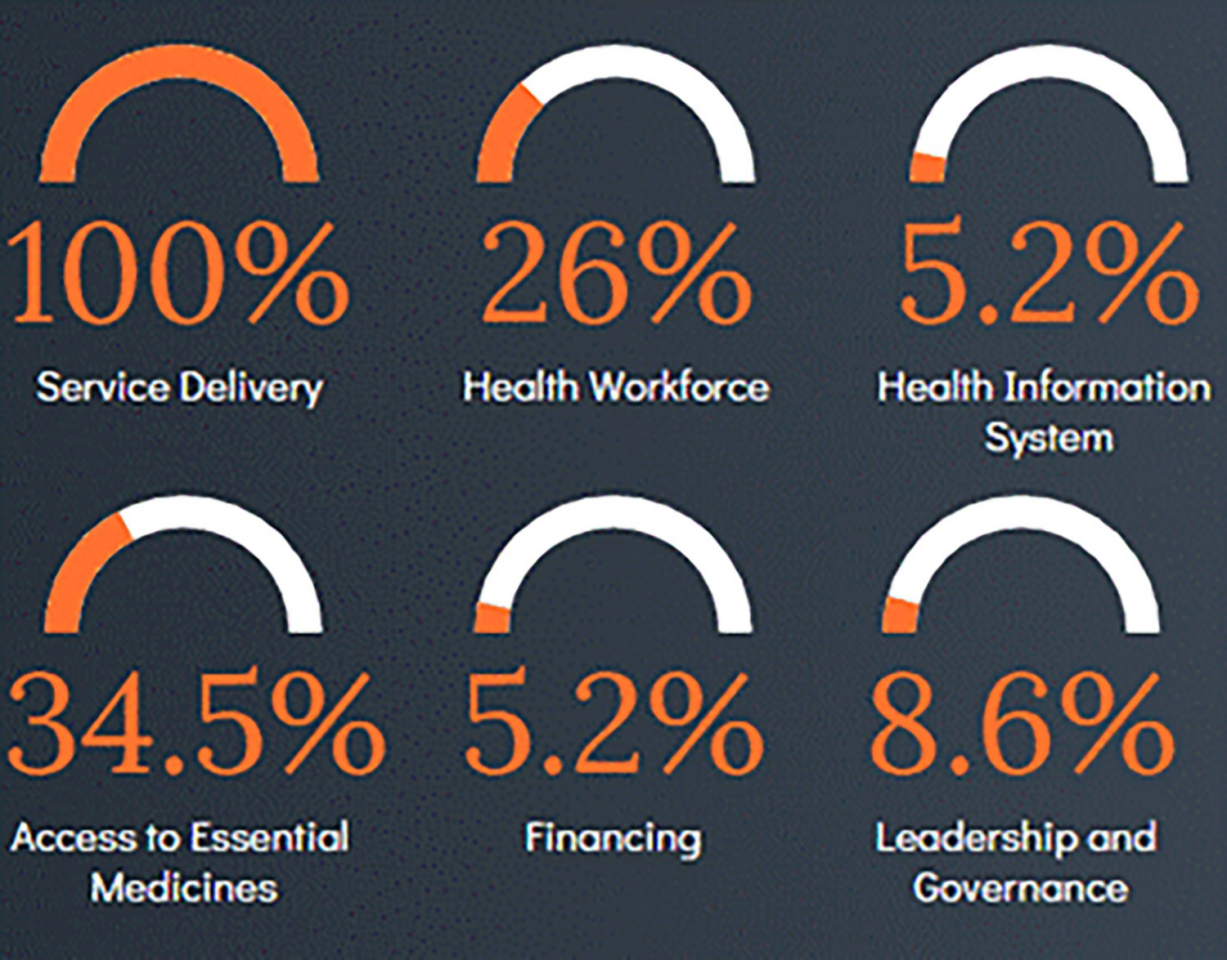
Proportion of included studies which addressed specific WHO health systems strengthening building blocks.

Using the health system strengthening categories, all 58 included studies had a focus on service delivery (Fig 2). However, the other building blocks were considered far less frequently. For instance, only five percent of included papers discussed health information systems or financing.

### 3.11. Strategies to improve routine childhood immunisation service delivery

One fifth of studies proposed strategies to improve routine childhood immunisation service delivery. Of these strategies, the five most commonly proposed are shown in Table 5.

**Table 5:** Most commonly proposed strategies from reviewed articles to improve routine childhood immunisation service delivery.

1. Community awareness campaigns 2. Maintain routine immunisation services during a pandemic in line with WHO guidelines 3. Catch-up activities 4. Strengthen the supply chain 5. Strengthen outreach services

## 4. Discussion

This review highlights that routine childhood immunisation services and subsequently routine childhood immunisation coverage and uptake globally have been significantly impacted by the COVID-19 pandemic. Included studies demonstrate widespread decline, not just in coverage but in the number of doses administered, service usage, and fixed and outreach immunisation sessions conducted across all five WHO regions examined. A quarter of the coverage-focused studies reported an overall decline in routine immunisation coverage and one fifth of the 58 included studies reported a decline in vaccine doses administered during the COVID-19 pandemic. Most studies discussed specific factors which contributed to changes in routine childhood immunisation coverage during the COVID-19 pandemic and proposed strategies that could be employed to improve routine immunisation. The majority of these strategies address access not acceptance related factors. When examining the included studies using the WHO health systems strengthening building blocks, all studies had a service delivery focus but less than 10 per cent discussed financing or health information systems.

A third of studies in this review addressed access to essential medicines, such as routine vaccines, during the COVID-19 pandemic. In May 2020, UNICEF called for support to clear the backlog in vaccine shipments from HICs where most vaccines are manufactured to LMICs for distribution [74,75]. This backlog was due to logistics challenges and increased transportation costs caused by the COVID-19 pandemic and caused a 70 to 80 per cent reduction in planned vaccine shipments [75]. This resulted in increased risk of stock-outs which has a direct impact on vaccine timeliness and number of doses that are able to be administered [75,76]. Key stakeholders in this area should prioritise the development of strategies to address these challenges so that these can easily be put in place if there is a future global pandemic.

During the pandemic there was a global shift in focus and resource allocation from routine health services to COVID-19 detection, prevention and treatment services [77]. This was more pronounced in settings where health systems were already overburdened and under-resourced [78]. Prior to the onset of the COVID-19 pandemic, these settings already had competing health system priorities, a limited health workforce and a greater burden of disease [79]. Focus and resource allocation needs to be shifted back to routine health service delivery. This is particularly important for low-resource settings where routine vaccination rates have significantly declined from pre-pandemic levels as outlined in this review.

Few papers in this review studied the impact of COVID-19 on EPI outreach services highlighting an evidence gap in this area. It is evident from these papers that outreach services have been impacted due to COVID-19 [18,22,37,38]. Outreach services are critical to vaccinate hard-to-reach populations. The Reaching Every District (RED) Strategy, developed by WHO for the African region in the early 2000s, focused on re-establishing routine immunisation outreach services to reach hard-to-reach populations to increase routine immunisation coverage [80]. Routine immunisation outreach is a key strategy in limited-resource settings to increase vaccination coverage. This reduction in EPI outreach sessions was coupled with the cancelling and postponement of planned vaccination campaigns. An analysis by Ho et al. of the Immunization Repository Campaign Delay Tracker which tracks COVID-19 related disruptions to mass vaccination campaigns for cholera, measles, meningitis, polio, tetanus, diphtheria, typhoid, and yellow fever at four times points from May 2020 to December 2021 found that mass vaccination campaigns across all vaccines were disrupted heavily by COVID-19 [81]. This reduction in services which targeted hard-to-reach populations has led to an increased risk in vaccine preventable disease outbreaks [81].

Studies which reported no significant impact on some aspects of routine immunisation may provide insight into strategies that work to continue childhood routine immunisation service delivery and uptake during a global pandemic [20,30,31,38]. Some of the strategies highlighted in these studies include the prioritisation of routine immunisation service delivery by the MoH, deploying health extension workers to health posts to provide community-based services including routine immunisation, a well-coordinated and centralised government risk communication strategy which decreed that critical essential health services continue and designated health facilities for vaccination to offset vaccination centres assigned as COVID-19 isolation centres.

This scoping review found that the WHO health systems strengthening building blocks of health information systems, financing, and leadership and governance were the least discussed across the studies. COVID-19 has further emphasised the need for strong health information systems to track and monitor disease and vaccine coverage and inform decision making and public health policy. Strengthening health information systems requires continuous efforts across all aspects of data collection, data management, data quality assessment, data analysis and data use [82]. Health information strengthening efforts undertaken during the COVID-19 pandemic should continue to be resourced and prioritised. The COVID-19 pandemic led to countries receiving increased amounts of external health financing to manage and deliver COVID-19 services [83]. The amount of external funding is likely to decrease as the pandemic becomes endemic. Re-directing funds and continuing to strengthen routine health service delivery will require ongoing supportive external and internal health financing policies. The importance of leadership and governance in improving routine immunisation services during the COVID-19 pandemic was discussed in only five of the studies. Frenk et al. note that this is the most complex challenge in health systems but one that must be invested in to ensure that health systems do not fail [84].

Most of the included studies were conducted in the African region, which may limit the generalisability of findings to other regions. One contributing factor is that 24 of the world’s 28 low income countries as classified by the World Bank are located in the African region [11]. Comparatively only 10 European countries are listed by the World Bank as LMIC, and this search did not identify any relevant studies from LMICs in Europe.

To strengthen routine childhood immunisation services and increase coverage in LMICs, a better understanding of what the impact has been, the key factors involved and strategies that could be employed is needed. As highlighted in Table 5, this should include a mixture of demand and supply strategies to ensure service availability and utilisation. However, the majority of strategies identified were focused on increasing supply and access. Future pandemics need to ensure that routine services such as vaccination continue to be prioritised, coupled with increased community awareness campaigns, to ensure that members of the public know they can still access these services and to build confidence and reiterate the importance of routine immunisation. Health system strengthening activities for routine childhood immunisation need to consider all the building blocks to ensure that along with service delivery and access to essential medicines, health workforce, health information systems, financing and leadership and governance are included.

To our knowledge this is the first scoping review on the impact of COVID-19 on routine childhood immunisation in LMICs looking at the period March 2020 to June 2022. Strengths of this review include using an authenticated scoping review methodology and conducting a systematic search across multiple databases and websites [85]. A limitation was restricting our search to English language publications only. However, this was rare with only four studies identified as non-English language. The time period of inclusion limits any consideration of the impact of COVID-19 vaccines and any associated vaccine hesitancy on routine immunisation uptake and acceptance.

We acknowledge that the LMIC classification is a limitation as it can have negative implications for research, policy and programming. We agree with Lencucha et al. that these classifications need to be adjusted to encompass a more nuanced approach to categorisation [8].

We also acknowledge that researchers in LMICs face multiple barriers to publishing their research as a limitation to this review and this needs to be a focus in the decolonisation of global health efforts. These researchers can often be marginalised and absent from the editorial process [86]. This is often coupled with working in an under-resourced setting where there is little time to analyse, report and publish data and a lack of available funding support. The lack of local funding support often means that local universities and non-governmental organisations seek funding from high income countries in partnership with researchers located in these high-income countries [87]. A review by Dimitris et al. found that although the number of LMIC-affiliated first and last author publications had quintupled from 2000 to 2017, a large amount of global health research using data collected in LMICs continues to come from high income countries [88]. Global health practitioners from HICs are critical to rectifying this imbalance.

## 5. Conclusion

This scoping review provides a comprehensive overview of the impact of COVID-19 on routine childhood immunisation across five WHO regions between 2020 and 2022. We found that the COVID-19 pandemic has significantly contributed to the decline in routine immunisation coverage reported and number of doses delivered in these regions. Vaccine timeliness has also been negatively impacted. This has likely led to a greater number of zero-dose and under-immunised children in these regions. Re-focusing efforts on improving routine childhood immunisation coverage is critical to child health and reducing the likelihood of vaccine preventable outbreaks in LMICs. This requires employing tailored, evidence-based, and context-specific strategies. Future studies examining the impact of COVID-19 on routine childhood immunisation may benefit from applying a WHO health system strengthening framework lens to understand the impact in each of these areas and develop specific strategies to address these findings.

## Supporting information

S1 Checklist PRIMSA checklist. (DOCX)

S1 Table Summary of the major findings of the 58 articles presented in a table format. (DOCX)

S2 Table Search strategy listing all terms used in the database searches. (DOCX)
