# Supplementary material for: Impact of COVID-19 on routine childhood immunisations in low- and middle-income countries: A scoping review
Source: PLOS Glob Public Health. 2023 Aug 23;3(8):e0002268. doi: 10.1371/journal.pgph.0002268 (PMC10446229; doi:10.1371/journal.pgph.0002268)
Supplement: S1 Table — (DOCX) [file pgph.0002268.s002.docx]

**Supplementary 1: major findings (N=58)**

| Authors | Country/ies | Aim | Study design | Study period | Comparison period | Major findings | Drop in coverage^[[1]](#footnote-1)^ or reduction in number of doses^[[2]](#footnote-2)^ delivered | | |
| --- | --- | --- | --- | --- | --- | --- | --- | --- | --- |
| Abid  (2022)[36] | Afghanistan | "Study the impact of the COVID-19 pandemic lockdown on the routine immunization coverage program in the province of Laghman, Afghanistan" | (comparative) repeated cross-sectional | April to July 2020 | April to July 2019 | Overall daily immunisation coverage declines of 21.4%. Declines were observed in all districts of Laghman province. Immunisation coverage reduced across all vaccine antigens studied with Hepatitis B the least affected and MCV and OPV4 the most affected. No gender differences in coverage were found. | Overall -21.4% (daily coverage) | | |
| Alves  (2021)[71] | Brazil | "Examine recent vaccination trends among Brazilian children during their first year of life, and the impact of the coronavirus disease 2019 (COVID-19) pandemic on these trends" | Repeated cross-sectional | January 2017 to February 2020 | March 2020 to December 2020 | There was no significant decrease in the number of immunisation doses per child between 2017 and 2020. Analysis showed no significant impact of COVID-19 isolation measures on the number of vaccines administered per child after March 2020, but the mean number of vaccinations was 10.6, which is lower than the 13 doses administered as per the immunisation schedule. | N/A | | |
| Babalola  (2022)[18] | Liberia | "Describe routine health service disruption and restoration strategies at 6 months into the epidemic in Liberia" | Mixed methods - qualitative /repeated cross-sectional | January to June 2020 | January to June 2019 | Themes included service disruption, fear of being diagnosed with COVID-19, fear of contracting COVID-19 at the health facility and fear that healthcare workers will inject them and their children with the COVID-19 vaccine. Routine immunisation services were offered by 92.7% of the study health facilities. Only 31.6% of the study health facilities provided outreach services from January to June 2020. Overall scheduled routine immunisation outreach activities conducted decreased by 47% in 2020 compared to 2019. The number of children vaccinated with BCG, Pentavalent1, Pentavalent3, and MCV decreased in 2020 compared to 2019. | BCG -34% (doses)  Pentavalent1 -47% (doses)  Pentavalent3 – 58% (doses)  Measles1 -79% (doses)  *June 2020 decreases as compared to June 2019 | | |
| Babatunde  (2022)[37] | Nigeria | "Look at the impact of the COVID-19 pandemic on routine immunization in Oyo State, Nigeria" | Repeated cross-sectional | July 2019 to August 2020 | N/A | Post-natal BCG fell by 3.7% and Hepatitis B birth dose by 3.5%. Vaccines administered at 14 weeks after birth also decreased. Average coverage reduced by 4.1% for Pentavalent3, 4% for OPV3, 3.1% for PCV3 and 1.6% for IPV. For yellow fever and MCV, the average coverage dropped by 12.4% and 15.9%. No clinic completed all of their planned fixed or outreach sessions in the post COVID-19 index case period. The average dropout rate pre COVID-19 index case was 5% and post-index case was slightly less at 4.7%. | BGC -3.7% (doses)  Hepatitis B -3.5% (doses)  Pentavalent3 -4.1% (doses)  OPV3 -4% (doses)  PCV3 -3.1% (doses)  IPV -1.6% (doses)  MCV -12.4% (doses)  Yellow fever -15.9% (doses) | | |
| Balogun  (2021)[19] | Nigeria | "Explore perspectives of health facility leaders in Lagos, Nigeria, on solutions and adaptations implemented to support maternal, newborn and child health service provision during the early phase of the COVID-19 pandemic" | Qualitative (key informant interviews) | July to November 2020 | N/A | Immunisation services continued to be provided in primary and tertiary facilities during the early phase of the COVID-19 pandemic. Fewer skilled doctors and nurses were allowed to come to work due to intra-facility transmission reduction measures. Most services appeared to return to normal after lockdown measures were eased in May 2020. Patient attendance reduced at many primary healthcare outpatient clinics and outreach services due to concern over risk of COVID-19 infection and difficulties accessing public transport. A catch-up immunisation strategy was employed for women who were unable to access routine immunisation services during lockdown. | N/A | | |
| Bekele  (2022)[20] | Ethiopia | "Assess maternal, newborn, and child health care utilization during the first six months of the COVID-19 pandemic, as well as potential barriers and enablers of service utilization from health care providers and clients" | Mixed methods - qualitative (framework theory approach)/ (comparative) repeated cross-sectional | March to August 2020 (quantitative), 2-20 November 2020 (qualitative) | March to August 2019 | Routine immunisation remained stable during the first six months of the COVID-19 pandemic. The main barriers to service utilisation identified by healthcare workers were fear of contracting COVID-19, increased transportation cost, travel restrictions. Patients stated fear of contracting COVID-19 and lack of transportation access as the main barriers. | N/A | | |
| Bello  (2021)[38] | Botswana, Eritrea, Eswatini, Ethiopia, Kenya, Lesotho, Madagascar, Malawi, Mauritius, Mozambique, Namibia, Rwanda, Seychelles, South Africa, South Sudan, Tanzania, Uganda, Zambia, Zimbabwe | "Identify the effects of COVID-19 on routine immunisation programme, surveillance, and morbidity and mortality rates in East and Southern African countries" | (comparative) repeated cross-sectional | January to August 2020 | January to August 2019 | Nine countries (47%) had decreased fixed sessions. Lesotho did not record any integrated supportive supervision visits in the 2020 period. Twelve (63%) countries had a decrease in outreach sessions planned and 7 (32%) the countries increased proportion of outreach sessions planned. Seven (37%) countries increased administrative coverage of DTP3, 10 (53%) experienced a drop in coverage and 2 (11%) maintained the same coverage. Six (32%) had increased MCV1 coverage while 13 (68%) had decreased coverage. Three countries (Kenya, Mozambique and Tanzania) had an increased proportion of fixed and outreach sessions and a corresponding increase in DTP2 and MCV1 coverage. | Botswana  Eritrea  Eswatini  Ethiopia  Kenya  Lesotho  Madagascar  Malawi  Mauritius  Mozambique  Namibia  Rwanda  **Seychelle  South Africa  South Sudan  Tanzania  Uganda  Zambia  Zimbabwe | *DPT3  -3%  -22%  +8%  0%  -29%  -32%  +10%  0%  +8%  +5%  +4%  +7%  +31%  +4%  -4%  +4%  +8%  0%  +5% | *MCV1  -8%  +13%  -18%  +1%  +33%  +3%  +4%  +4%  -12%  +11%  -2%  -1%  -11%  -2%  -20%  -2%  -3%  -5%  -11% |
|  |  |  |  |  |  |  | *Coverage  ** high-income country | | |
| Bhadoria  (2021)[62] | India | "Assess the overall trend and evaluate the vaccination coverage during COVID-19 pandemic in a tertiary care hospital in Gwalior district" | Repeated cross-sectional | February 1, 2020, to August 31, 2020 | N/A | Fifty-one (6.2%) children had delayed vaccination. This was observed for Pentavalent2 (43.1%), Pentavalent3 (51%) and MR1 (5.9%). Vaccination delay is significantly associated with monthly distribution of children reporting for vaccination. Maximum delay was seen in June 2020 (20.2%), followed by May (18.5%). This may be due to the closed immunisation outpatient department in April. | N/A | | |
| Bimpong  (2021)[21] | Ghana | "Examine the impact that the pandemic had on childhood vaccination services at the Tamale Teaching Hospital" | Mixed - qualitative (in-depth interviews and focus groups)/(comparative) repeated cross-sectional | March 1, 2020, to February 28, 2021 | March 1, 2019, to February 29, 2020 | General decline in all vaccines included in the study except for MR2. The greatest average decline was in BCG birth dose (47%) and the least in MR1 (10.5%). The decline increased with age at vaccination. Overall decline in vaccinations was 38.3% when compared to the pre-pandemic year. Those interviewed stated there was a reduction in mothers bringing their children for immunisation due to fear of COVID-19 infection and misinformation. | Overall -38.3% (average doses)  BCG -47% (average doses)  Pentavalent1 -42% (average doses)  Pentavalent2 -40.3% (average doses)  Pentavalent3 -39% (average doses)  MR1 -10.5% (average doses)  MR2 +0.9% (average doses) | | |
| Burt  (2021)[57] | Uganda | "Assess the impact on maternal, neonatal, child, sexual and reproductive health services from July 2019 to December 2020 at the Kawempe National Referral Hospital in Kampala, Uganda" | Repeated cross-sectional | July 2019 to March 2020 | April to June 2020 (lockdown period)  July to December 2020 (post-lockdown period) | There was a significant decrease in polio, tetanus, diphtheria, Hepatitis B, Hib, rotavirus and PCV immunisations for newborns. Since the end of lockdown, no change was found in the rate of children receiving BCG at birth, OPV, PCV or Rotavirus vaccines. However, there were 960 fewer monthly attendances for routine immunisation services after the end of lockdown. Measles vaccinations increased, however this was due to a catch-up campaign after a long stock-out of the vaccine. | N/A | | |
| Chandir  (2020)[58] | Pakistan | "Examine the impact of COVID-19 lockdown on routine immunisation in Sindh province, Pakistan" | Ecological | March 23, 2020, to May 09 2020 | September 23, 2019, to March 22 2020 | From September 23, 2019, to March 22, 2020, there were 2,463,595 immunisation visits of which 31.9% were children registering for the first time. During lockdown from March 23 to May 9, 2020, there were 323,855 immunisation visits 25.7% of which were children registering for the first time. Only 30.7% of children due for follow-up immunisations were vaccinated during lockdown. Overall, there was a 51% decrease in the average daily immunisations during lockdown compared to the baseline period in 2019. There was limited outreach during lockdown. Rural areas were more affected by lockdown than urban areas. There was a steady recovery of coverage rates after lockdown was lifted with absolute improvement of 9% in average daily immunization visits from May 10 to July 11, 2020. | Overall -51% (doses) | | |
| Chelo  (2021)[49] | Cameroon | "Assess the impact of the COVID-19 pandemic on the uptake of consultation and immunisation services in a level-3 referral paediatric hospital in Yaounde, Cameroon" | Repeated cross-sectional | January 1, 2016, to May 31, 2020 | N/A | From January 1, 2016, and May 31, 2020, 5,522 BCG, 6,034 DTP3, and 5,435 MCV doses were administered. Already in decline, the demand for the BCG vaccine further declined from the beginning of the pandemic in March 2020 with a resurgence in May 2020. All vaccines were impacted by the COVID-19 pandemic, however this was less so for vaccines given close to birth (BCG and OPV0) than for other vaccines in the EPI. | N/A | | |
| Colome-Hidalgo  (2022)[39] | Dominican Republic | "Assess the impact of the COVID-19 pandemic on routine infant vaccination in the Dominican Republic" | Ecological | 2020 | 2019 | Coverage for all vaccines decreased by 10.4 percentage points (pp) from 2019 to 2020. Coverage for Pentavalent3 decreased from 90.1% in 2019 to 81.1% in 2020. BCG had the highest coverage (99.2%) and the second largest decrease after MMR. Hepatitis B had the lowest coverage (71.4%), and the rotavirus vaccine showed the lowest decrease. The number of partially vaccinated children increased by 66% and unvaccinated children by 37.6%. The national drop-out rate increased by 1.1% in 2020 which was directly proportional to the number of COVID-19 cases per month. | Overall -10.4pp (coverage)  BCG -13.8pp (coverage)  Hepatitis B -9.2pp (coverage)  Rotavirus -3.3pp (coverage)  Polio -11.6pp (coverage)  Pneumococcus -11.9pp (coverage)  MMR -14pp (coverage)  Pentavalent1 -13pp (coverage)  Pentavalent3 -9pp (coverage) | | |
| Connolly  (2022)[72] | Haiti, Lesotho, Liberia, Malawi | "Examine changes in vaccination of children younger than 1 year during the COVID-19 pandemic in Haiti, Lesotho, Liberia, and Malawi" | (comparative) repeated cross-sectional (modelled data) | March 2020 - April 2021 | March 2020 - April 2021 (modelled expected counts assuming no pandemic using data from January 2016 to February 2020, except for Haiti, which started from January 2017) | No country site had a statistically significant decline in vaccines administered at birth in the period March to August 2020. Haiti, Lesotho and Liberia had a statistically significant decline in MCV early in this period. From September 2020 to February 2021, vaccination numbers for OPV, IPV, Pentavalent and PCV and the MCV decreased in Lesotho. BCG vaccination decreased in Haiti and BCG, Pentavalent, IPV/OPV and PCV remained as expected or decreased in Malawi. From March to August 2021 Liberia's vaccination levels remained at predicted levels except for a sharp decrease in March and April. Coverage in Malawi, Haiti and Lesotho returned to almost expected levels or above for most vaccines. For the period March 2020 to August 2021 all countries except Liberia had a cumulative deficit. Birth dose vaccines were least affected. | N/A | | |
| da Silva  (2021)[50] | Brazil | "Analyse the number of doses of vaccine against MMR applied before and after the beginning of mitigation measures due to COVID-19 pandemic in Brazil" | Ecological | April to September 2020 | April 2019 to March 2020 | The number of MMR vaccine doses administered between April 2020 and September 2020 was considerably lower than from April 2019 to March 2020. A possible reason for this is the COVID-19 restrictions. Three of the five regions in the country showed a statistically significant reduction in the median number of MMR doses applied while public health emergency measures were in place. Out of 27 states, seven had a statistically significant reduction. The median number of doses reduced by 43%. | MMR -43% (median number of doses) | | |
| Dabo  (2020)[40]  (pre-print) | Guinea | "Assess the early impact of COVID-19 on vaccine activities by comparing current trends to trends over the past year when vaccine coverage of major antigens (BCG, OPV, DTP-Hepatitis B-Hib, MMR, IPV, and Td) had improved considerably." | Ecological | January to June 2020 | January to December 2019 | COVID-19 caused a significant interruption of the vaccination program for all vaccines at the national and district level. This interruption caused significant delays for IPV, DTP1, DTP2, DTP3 and adeno-associated virus (AAV). | N/A | | |
| Doubova  (2021)[34] | Mexico | "Estimate the overall effect of the pandemic on essential health service use and outcomes in Mexico, describe observed and predicted trends in services over 24 months, and to estimate the number of visits lost through December 2020" | repeated cross-sectional | April to December 2020 | January 2019 to March 2020 | Overall vaccinations declined by 36%. Declines for BCG, Pentavalent3, rotavirus and PCV ranged from 28% (rotavirus) to 46% (Pentavalent3). There was no statistically significant decline in the number of children receiving MMR2. | Overall -36% (doses)  Pentavalent3 -46% (doses)  Rotavirus -28% (doses) | | |
| Enbiale  (2021)[32] | Ethiopia | "Assess the effect of preventive COVID-19 measures on essential healthcare services in selected health facilities of Ethiopia" | (comparative) repeated cross-sectional | March 9 to July 6, 2020 | July 7, 2019, to March 8, 2020 | The mean number of children that received full immunisation did not change. Average childhood immunisation in April 2020 decreased by 11% from the annual averages. In August 2020 the average was higher than the pre-pandemic period by about 11%. | Overall -11% (average doses in April 2020)  Overall +11% (average doses in August 2020) | | |
| Gupta  (2021)[63] | India | “To study the impact of lockdown and reasons for this impact on vaccination” | cross-sectional | 2021 | June 10, 2020, to July 9, 2020 | One third of children had a delay in their first vaccination of 1-4 weeks, 23.66% a one-to-three-month gap, 17.20% a one-week gap and 13.36% no gap post lockdown. Fear of COVID-19 was the most reported reason (50.44%) for the delay. For those who had more than a six-month delay, 55% forgot, 29% were afraid of COVID-19 and 14% had family problems. These were predominantly children aged 5, 10 and 16. For the second scheduled dose post lockdown 94.74% had less than one month delay stating family problems (43.75%), fear of COVID-19 (31.25%) and sick child (25%) as the reason. For the third scheduled dose post lockdown 89.29% delayed less than one week. | N/A | | |
| Hanifi  (2022)[22] | Bangladesh | "Investigate the impact of COVID-19 on child routine immunization in a rural area of Bangladesh and consider the broader implications" | (sequential) mixed methods - (comparative)cross sectional/qualitative (in-depth interviews) | March 1 to May 31, 2020 | March 2019 to May 2020 | In 2019 99% of planned monthly EPI outreach sessions were carried out in the Chakaria Health and Demographic Surveillance System area. In 2020 EPI outreach declined markedly to 74% in March, 10% in April and 3% in May. From March to May 2020 71% of the EPI outreach sessions were suspended and 2,116 children missed their routine vaccines. This was due to the unwillingness of villagers to hold EPI outreach sessions, absent vaccinators, social distancing recommendations, lack of personal protective equipment (PPE) and not receiving guidelines to continue vaccination sessions. | N/A | | |
| Hategeka  (2021 pre-print)[73] | DRC | "Evaluate the impact of COVID-19 and its related response measures on the use of health services in Kinshasa during the first wave of the pandemic (March-September) to provide insights to inform the ongoing response and future infectious disease outbreaks" | repeated cross-sectional | March to December 2020 | January 2018 to February 2020 | Overall COVID-19 did not significantly affect vaccinations administered. | N/A | | |
| Jain  (2021)[41] | India | "Use primary survey data to examine disruptions to child immunizations during the COVID-19 lock down and the extent to which the government's efforts at catch-up immunization after the lockdown was eased were successful at reaching missed children in low-income households in Rajasthan, India" | cross-sectional | August 2020 to October 2020 | "unexposed": Children who turned 12 months before March 2020  "partially exposed": Children who turned 12 months between March and May 2020  "heavily exposed": Children who turned 12 between June and August  "post-exposure": Children who turned 12 in September or October 2020 | Children that were due their immunisations during lockdown experienced a shift in the timing of their MCV1 vaccine compared to children who were due before lockdown and after lockdown. There was no difference in timeliness of MCV between pre and post lockdown children. Coverage prior to lockdown for children who turned 12 months old in February 2020 or earlier was 74.5%. This dropped to 70.4% for children partially in the lockdown period and 64.1% for children in the lockdown period. This increased to 71% after lockdown ended. The probability of completed first year immunisation was higher among children with an immunisation card pre, during and post pandemic. For pre-pandemic children the probability of first year immunisation statis was lower among those less educated, poorer, and lower caste households. | Overall -4.1pp (doses for children partially in lockdown period)  Overall -10.4pp (doses for children in the lockdown period)  Overall -3.5pp (doses for children after lockdown had ended) | | |
| Kassie  (2021)[33] | Ethiopia | "Evaluate the early indirect impact of COVID-19 on the utilisation of reproductive, maternal, and newborn health services at government health facilities in South West Ethiopia, and its consequences" | (comparative) repeated cross-sectional | March 2020 to June 2020 | March 2019 to June 2019 | Overall newborn immunisation service utilisation decreased by 28.5%. Service utilisation varied across zones with a 48.2% reduction in Bench Sheko, 35.6% in Keffa, 26.4% in Sheka and 11.9% in West Omo. Health centre services were reduced by more than one-third while the hospital services were reduced by more than 50%. | N/A | | |
| Khan  (2021)[48] | India | "Describe the impact of COVID-19 pandemic on immunisation in a tertiary level health-care facility" | (comparative) repeated cross-sectional | 1 January to 31 July 2020 | 1 January to 31 July 2019 | A significant decline in vaccinations administered as part of the Universal Immunisation Program for all antigens given to children under five years except Hepatitis B birth dose. The most significant drop in vaccinations administered was observed in April 2020. | Overall -17.7 percent change (doses)  BCG -27.7 percent change (doses)  Pentavalent1 -21.9 percent change (doses)  Pentavalent3 -53.6 percent change (doses)  MR1 -13.1 percent change (doses)  MR2 -35.3 percent change (doses) | | |
| Khan  (2022)[64] | Pakistan | "Understand the impact of COVID-19 pandemic on routine immunization uptake in children in Islamabad, Pakistan" | repeated cross-sectional | November 2020 to January 2021 | N/A | Among the enrolled children 34.5% had delayed vaccination. Children were more likely to be vaccinated at a private centre. Factors significantly associated with vaccination status were maternal education and place of delivery. Lockdown and closure of the EPI vaccination centres were the main reported cause for vaccination delays. | N/A | | |
| Khatiwada  (2021)[23] | Nepal | "Explore the challenges and experiences of providers and users of childhood immunisation services in Nepal during the COVID-19 pandemic" | qualitative (semi-structured interview) | August 2020 to December 2020 | N/A | Most participants believed that the pandemic had impacted childhood immunisation service delivery and utilisation. All study participants expressed fear and anxiety due to the COVID-19 pandemic. Service providers were motivated to continue to provide services by their family members and moral obligation. Prior health education on the importance of immunisation motivated caregivers to bring their children for vaccination during the pandemic. Participants reported a lack of PPE. Service providers reported that there were no clear guidelines on service delivery during the pandemic, lack of adequate resource support from local government, lack of PPE and a lack of a proper database tracking on health care services utilisation. Service providers reported that vaccine logistics were only impacted at the beginning of the pandemic. | N/A | | |
| Kinikar  (2021)[51] | India | "Identify the magnitude of decline in routine paediatric vaccine delivery at a public tertiary care teaching hospital" | comparative cross-sectional | 1st January to 13th May 2020 | Period 1: 1st January 2020 to 24th March 2020  Period 2: 25th March to 13th May 2020 | There was some decline in the number of newborn immunisations (BCG, OPV, Hepatitis B birth dose) in hospital during lockdown but this was not statistically significant compared to pre-lockdown. There was a statistically significant decline in the number of vaccines given in infancy and children under five (Pentavalent, rotavirus, OPV, IPV, DTP booster) during lockdown compared to pre-lockdown. For the MR vaccine, there was a statistically significant decline in the number of children immunised in lockdown compared to pre-lockdown. | N/A | | |
| Kotiso  (2022)[59] | Yemen | "Evaluate the impact of COVID-19 on the continuity of the health services provision at public hospitals in Yemen" | comparative repeated cross-sectional | January 2020 to June 2020 | January 2019 to June 2019 | From April to June 2020 there was a significant reduction in Pentavalent3 service utilisation compared to the same period in 2019. There was no significant reduction in February or March 2020 compared to the same period in the previous year. | Pentavalent3 -6.7% (doses) | | |
| Mahfouz  (2021)[65] | Egypt | "Assess awareness, commitment, and adherence to compulsory immunization schedule during COVID-19 lockdown in Egypt" | cross-sectional | June 2018 to June 2020 | N/A | Of the children in the study 93.7% received the Hepatitis B vaccine and 96.3% the BCG vaccine on time. Of children eligible for mandatory vaccines at 2, 4 and 6 months 82.5% were vaccinated. A third of children did not receive their mandatory booster dose at 18 months. A primary healthcare centre was the most common place for vaccination. Approximately 23% of children who missed a vaccine preferred to postpone until after the outbreak ended. Fear of contracting COVID-19 was given by 27.2% as the reason for not having vaccine. Approximately 80% of parents said that it was important to catch-up any delayed vaccinations. Participants in cities showed better adherence to the vaccination schedule at 2, 4, 6 and 12 months than those in villages. | N/A | | |
| Mansour  (2021)[60] | Lebanon | "Assess the changes in the utilisation of routine immunisation services in both the public and private sectors following the COVID-19 pandemic" | cross-sectional | October 2019 to April 2020 | October 2018 to April 2019 | Of the physicians surveyed, 77.4% reported an overall decrease in the utilisation of routine immunisation services between October 2019 and April 2020. Overall, the utilisation of vaccination services decreased by 31% nationally. This was estimated to be greater in the private sector at 46.9%, compared to a 20% decline in the public sector. Percent change in reduction rates was 57.5% for OPV, 53.3% for PCV, 53.3% for MCV, and 49.9% for MMR vaccines. | N/A | | |
| Manzoor  (2022)[66] | Pakistan | "Determine the impact of COVID-19 pandemic on routine immunization of children in Pakistan" | cross-sectional | March to September 2021 | N/A | Most parents surveyed agreed that children should be vaccinated and believe it is essential for child health. Eighty percent of parents had a scheduled vaccination for their child during COVID-19 and 18% delayed vaccination during this time. Only 2% of parents reported that their children had no routine immunisations during the pandemic. The main reason for delayed vaccination was fear of contracting COVIS-19. The preferred location for vaccination service delivery was at home (40%) followed by at a dedicated hospital (36%). | N/A | | |
| Masresha  (2020)[52] | Angola, Central African Republic (CAR), Chad, DRC, Gabon, Guinea, Nigeria, South Sudan, Burundi, Eritrea, Ghana, Kenya, Rwanda, Senegal and Tanzania | "Examine the actual routine immunization program performance in selected countries in the African Region by comparing the number of children vaccinated in the early months of the COVID-19 pandemic to the number vaccinated in the months prior to the arrival of COVID-19 in the countries" | repeated cross-sectional | January 2018 to June 2020 | N/A | Overall, the number of children vaccinated with DTP1, DTP3 and MCV1 declined in April and May 2020 compared with the first quarter of the year. The number of children vaccinated with DTP3 and MCV1 increased in June compared with April and May. Thirteen out of the 15 countries showed a monthly average decline in the number of vaccine doses provided with six countries having more than a 10% decline. The monthly mean of recipients of first dose measles vaccine was lower in nine countries in the second quarter of 2020 compared to the first. | Angola  CAR  Chad  DRC  Gabon  Guinea  Nigeria  South Sudan  Burundi  Eritrea  Ghana  Kenya  Rwanda  Senegal  Tanzania | *DPT3  -12%  -3%  6%  1%  -28%  -52%  -12%  -7%  -12%  -9%  -4%  -2%  -2%  -14%  -3% | *MCV1  6%  -3%  13%  2%  -40%  -53%  -13%  9%  -20%  2%  -4%  10%  -4%  -5%  0% |
|  |  |  |  |  |  |  | *percent change doses | | |
| Miretu  (2021)[89] | Ethiopia | "Assess the impact of COVID-19 on vaccination coverage among children aged 15 to 23 months in Dessie town, from July 22- August 7, 2020" | cross-sectional | July 22 to August 7, 2020 | N/A | Using vaccination card plus recall, 57.4% of children had finished all recommended vaccines. During the COVID-19 outbreak, age-eligible vaccination was 12.5% lower than pre-pandemic. Having a mother as the main caregiver, a mother/caregiver who can read and write, a mother/caregiver who had been educated from grade 1 to 8, a mother/caregiver who knows the benefit of vaccination, a mother/caregiver who was married, and being less than 30 minutes from a health facility were all predictive of a child receiving their full vaccination schedule. | N/A | | |
| Mongbo  (2021)[29] | Benin, Burkina Faso, Cote d'Ivoire, Guinea, Mali, Mauritania, Niger, Senegal, Togo | "Analyse the challenges and solutions for maintaining the continuity of essential health services during the COVID-19 pandemic in Francophone West Africa" | cross-sectional | April 2020 | N/A | Respondents reported that a lack of framework, misinformation about availability of services, community and provider fears and non-compliance with physical distancing at health centres and during mass vaccination campaigns, stock shortages, and human resource constraints meant it was challenging to continue to provide essential services. Respondents recommended the provision of PPE for healthcare workers, reorganising service delivery and increasing the use of telehealth, digital health, and social media. | N/A | | |
| Moreno-Montoya  (2021)[42] | Colombia | "Assess the impact of the COVID-19 pandemic on routine childhood vaccination coverage in Colombia by age group, rural/urban residence, state and vaccine type" | ecological | March 2020 to October 2020 | March 2019 to October 2019 | Overall decline in vaccination coverage of 14.4% from 2019 to 2020. The greatest reduction in proportion vaccinated was in children <12 months for PCV2. The proportion of children aged 12-23 months declined for yellow fever by 16.4%. For children aged five, OPV2 decreased the most with a difference of 11.4% between 2019 and 2020. There was a statistically significant effect on vaccine coverage in rural areas compared with urban areas for children <12 months and 5 years of age. | Overall -14.4% (coverage)  Yellow fever -16.4% (coverage)  OPV2 -11.4% (coverage) | | |
| Moura  (2022)[43] | Brazil | "Assess the impact of the COVID-19 pandemic on childhood vaccination coverage by Brazil's Sistema Único de Saúde system" | repeated cross-sectional | February 2020 to December 2020 | January 2015 to February 2020 | From 2019 to 2020 Hepatitis B, MMR, Meningococcal and BCG experienced the largest decrease in vaccine coverage of between 20-32%. Hepatitis A and IPV dropped by 10% from 2019 to 2020. Influenza, DTP, yellow fever, and Pentavalent increased in 2020. In the North of Brazil, the poorest region, vaccine delivery declined outside of the forecasted ranges earlier in 2020 but subsequently rebounded. In Brazil’s wealthiest regions, initial vaccine delivery declined and remained well below forecasted rates through to the end of 2020. | Hepatitis B -31.89% (dose distribution)  MMR -27.26% (dose distribution)  Meningococcal -25.02% (dose distribution)  BCG -20% (dose distribution)  Hepatitis A -15.14% (dose distribution)  IPV -10% (dose distribution) | | |
| Murthy  (2022)[67] | India | "Examine the impact of a phone-based intervention on immunization uptake in Special Neonatal Care Unit (SNCU) babies" | cross-sectional | June to September 2020 | N/A | Data collected from the initial call showed 65.2% babies were fully immunised and 34.1% had missed at least one scheduled vaccine. Data collected from the first follow-up call showed that 42% of families had vaccinated their baby and 42% had not. The immunisation rate increased by 22% from the initial call to 79.5%. Data from the second follow-up call showed an additional 44.1% of babies had been vaccinated and 39% were not vaccinated. | N/A | | |
| Nguyen  (2021)[53] | Bangladesh | "Examine the changes to health and nutrition service delivery and utilisation in urban Bangladesh during and after enforcement of COVID-19 restrictions and identify adaptations and potential solutions to strengthen delivery and uptake" | repeated cross-sectional | September to October 2020 | February 2020 | Child immunisations declined by 38 percentage points and health facility visits for immunisation by 61 percentage points. The most reported challenges in service utilisation were increased workload during the pandemic (56%), being scared to delivery services at home (38%) and lack of transportation to reach health facilities (29%). The most reported challenges for pregnant women and mothers were fear of leaving the house (33% and 19%) and getting infected with COVID-19 at the health centre (28% and 14%). More than half of health providers reported that they adapted child immunisation services. This included coordinating with their colleagues (48%) or supervisors (16%) to arrange immunisation drives, calling mothers to schedule immunisation appointments (36%), and sending reminders to mothers via WhatsApp messages or phone calls to encourage them to adhere to immunisation schedules (16%). | Overall -38 percentage points (doses) | | |
| Patel  (2022)[90] | India | "Explore enablers and barriers to help in formulating appropriate strategies for ensuring uninterrupted routine immunisation services applicable for a similar context" | cross-sectional | June to August 2020 | N/A | The major enablers to providing routine immunisation services identified were issuing identification cards, provision of PPE and sanitizer, vaccine supply and related logistics, community support, parents' concern for their child's immunisation, family and peer support. The major barriers to providing routine immunisation services identified were limited supply of PPE, unavailability of vaccines, deployment to COVID-19 activities, challenges with community mobilization, lack of COVID-19 awareness, transport restrictions, and pandemic associated stress and anxiety. | N/A | | |
| Powelson  (2022)[24] | Mozambique | "Describe caregivers' immunisation experiences and identify determinants of vaccine dropout" | qualitative (in-depth interviews) | February 2020 and March 2021 | N/A | Partially vaccinated children were missing an average of 5.1 vaccines. The most missed vaccines were IPV and MR. Four main patterns of barriers leading to dropout were identified: 1) social norms and limited family support place the immunisation burden on mothers, 2) perceived poor quality of health services reduces caregiver trust in vaccination services, 3) concern about side effects causes vaccine hesitancy, and caregivers hesitate to seek and 4) advocate for vaccination due to power imbalances with health workers. Facilitators identified by the caregivers of fully vaccinated children were accompaniment to health facilities and assistance caring for other children. Other barriers identified by caregivers and healthcare providers include fear of contracting COVID-19, vaccine stock-outs, lack of PPE supplied such as gloves and inability to social distance at the facility. | N/A | | |
| Procianoy  (2022)[44] | Brazil | "Evaluate the impact of the COVID-19 pandemic on the vaccination numbers for immunization geared toward individuals under 12 months of age in Brazil" | ecological | 2013 to 2020 inclusive | N/A | Nine out of ten vaccines included in this study recorded the lowest historical numbers of vaccination coverage in 2020. The Pentavalent vaccine was the only one which did not record the lowest historical number. Of these nine, all fell by at least 9% and BCG, Hepatitis B, PCV10 booster dose and MMR1 fell by more than 14%. The biggest decline was seen in Hepatitis B vaccine at 20.4%. Overall vaccination coverage declined by 11.10% from 2019 to 2020. | Overall -9% (coverage)  Hepatitis B -20.4% (coverage) | | |
| Rahman  (2021)[45] | Pakistan | "Analyse the effect of COVID-19 pandemic on routine immunisations practice in Pakistan" | retrospective cohort study | April 1, 2020, to the end of January 2021 | November 1, 2019, to the end of March 2020 (Pre-lockdown: November 1, 2019, to March 31, 2020  Lockdown: April 1, 2020, to August 31, 2020  Post-lockdown: September 1, 2020, to January 2021) | During lockdown immunisation coverage declined between 30.7% to 48.6% compared to the pre-lockdown period. After the first lockdown there was only a small decline between 2.8% and 20.2%. MCV coverage significantly decreased during the COVID-19 lockdown (40.7% for first dose and 48.6% for second dose). After lockdown monthly vaccination decreased by 17.8% for measles first dose and 20.2% for second dose. Rotavirus vaccination dropped by more than 30% during lockdown and 15% post lockdown. Pentavalent vaccine decreased by 33% for first dose, 35% doe second dose and 37% for third dose during lockdown. This changed to 13%, 10% and 5% respectively after lockdown. For polio and PCV there was a 31% decrease for first dose, 33% for second and 35% for third in lockdown compared to pre-lockdown. | **During lockdown (coverage)**  MR1 -40.7 percent change  MR2 -48.6 percent change  Rotavirus -30 percent change  Pentavalent1 -33 percent change  Pentavalent2 -35 percent change  Pentavalent3 -37 percent change  Polio +PCV1 -30.7 percent change  Polio+PCV2 -33.6 percent change  Polio+PCV3 -35 percent change  **Post lockdown (coverage)**  MR1 -17.8 percent change  MR2 -20.2 percent change  Rotavirus -15 percent change  Pentavalent1 -13 percent change  Pentavalent2 -10 percent change  Pentavalent3 -5 percent change  Polio +PCV1 -12.3 percent change  Polio+PCV2-9.6 percent change  Polio+PCV3 -2.8 percent change | | |
| Rizwan  (2021)[68] | Pakistan | "Determine the impact of COVID-19 pandemic on vaccination of the children under two years of age" | cross-sectional | 25 July 2020 to 7 August 2020 | March - December 2020  Stay-at-home (March-June) Reopening (July-December) | During the pandemic 45.8% of the 345 children completed their vaccination, 33.9% delayed their vaccination and 20.3% missed their vaccination. For specific vaccines 4% missed BCG and OPV at birth, 9% missed first dose and 6% missed second dose of PCV, Pentavalent, rotavirus and OPV. Third dose of PCV, Pentavalent, rotavirus, OPV and IPV was missed by 4.6%. First and second dose measles was missed by 3.4%. Fear of contracting COVID-19 and transportation issues during lockdown were the most common reasons for delay. Factors associated with missing vaccination were father's lower educational status, low monthly income and distance from the vaccination centre. | N/A | | |
| Santos  (2021)[54] | Brazil | "Assess the impact of the COVID-19 pandemic on routine paediatric vaccinations in the country" | (comparative) repeated cross-sectional | January 2010 to December 2020 | March - December 2020  Stay-at-home (March-June) Reopening (July-December) | Vaccine doses administered declined during the lockdown period. For children aged 0 to 2 years the North recorded the highest decline followed by the Northeast and the Central-West. For children aged >2 to 6 years the North recorded the highest decline. The number of doses administered in the reopening period increased in all regions. The number of measles-containing vaccines did not recover at the same rate. Poorer regions were more severely affected. | N/A | | |
| Sato  (2021)[55] | Nigeria | "Evaluate the impact of COVID-19 lockdown on the vaccination service delivery in Nigeria" | repeated cross-sectional | January to September 2020 | N/A | The lowest number of vaccinations were administered in May 2020 for all vaccines. This number returned to pre-lockdown levels in June 2020. Vaccinations administered in three northern states, North Central, North East and North West declined in May 2020 while Southern states observed only a minor decline in April 2020 or no decline. | N/A | | |
| Shapira  (2021)[28] | Cameroon, Liberia, Mali, Sierra Leone, DRC, Malawi, Nigeria, Somalia | "Quantify the disruption of maternal and child health services during the COVID-19 pandemic using nationally comprehensive administrative data in eight sub-Saharan African nations" | repeated cross-sectional | March 2020 to July 2020 | January 2018 to February 2020 | For all countries except the DRC, the number of children who received the third dose Pentavalent vaccine dropped for at least one month. The cumulative reduction in the March to July 2020 period ranged from 2% in Cameroon to 17% in Mali. The biggest disruptions were in April and May 2020. These continued in June and July for Mali, Nigeria and Sierra Leone. The number of vaccinated children is not significantly different from pre-COVID-19 levels by June 2020 in Liberia and Somalia. There is a similar pattern for BCG vaccinations administered but with smaller reductions on average. Three of the seven countries reporting a significant shortfall in total BCG vaccines administered. | N/A | | |
| Shapiro  (2022)[69] | Middle-income (Brazil; China; India; Indonesia; Malaysia; Mexico; Philippines; Thailand; and Vietnam) and high-income (Australia; Canada; Denmark; Finland; France; Germany; Hong Kong; Italy; Norway; Saudi Arabia; Singapore; Spain; Sweden; Taiwan; United Arab Emirates; United Kingdom; and United States of America) | "Assess the extent of and reasons for missed or delayed vaccinations in middle- and high-income countries in the early months of the pandemic" | cross-sectional | 14 May 2020 to 9 June 2020 | N/A | Households in middle-income countries reported more missed childhood vaccination (7.6%) than high-income countries (3%). COVID-19 risk factors, younger age, male sex, employment, psychological distress, larger household size and more children correlated with missed childhood vaccination in middle-income countries. The most common reasons stated for missed vaccination were fear of contracting COVID-19 at the vaccination clinic (15%) or when leaving the house (11%). Children were more likely to miss vaccinations in middle-income countries due to cost, movement restrictions, save services for others who needed them, fear of contracting COVID-19. In both middle- and high-income countries children were more likely to have missed a vaccine due to clinic closure of worry about giving COVID-19 to non-adults. | N/A | | |
| Shet  (2021)[61] | India | "Assess the degree of disruption to vaccination services, explored barriers to healthcare provision and identified innovative ways to regain losses in vaccination coverage" | repeated cross-sectional | April to June 2020 and September 2020 | N/A | One-third of respondents reported complete or partial suspension of immunisation services at their respective centres. In survey 1 from April-June 2020 vaccination services dropped by half in 83.1% of centres and by 32.6% in survey 2 from September 2020. Vaccine campaign interruption for MR and polio was reported by 37.7% of respondents. Only 38.7% of respondents reported having a catch-up vaccination plan for when restrictions eased. Supply-side barriers include limited healthcare worker availability, financial constraints, limited supplies such as PPE. Barriers to caregiver vaccine uptake included low awareness of service availability, transportation limitations, fear of contracting COVID-19 in clinical settings and financial constraints. Almost two-thirds of respondents were aware of safe provision of vaccination during COVID-19 guidelines but 42.5% were unaware of the national vaccination catch-up programmes. | N/A | | |
| Shikuku  (2020 pre-print)[30] | Kenya | "Determine the initial impact of COVID-19 pandemic on reproductive, maternal, newborn, child, and adolescent health services in Kenya" | (comparative) repeated cross-sectional | March 2020 to June 2020 | March 2019 to June 2019 | No significant change in the mean total immunisation service hospital attendance per month from March to June 2020 compared with the equivalent four-month period in 2019. Data showed a reduction in Pentavalent immunisations in April 2020 and a sustained increase in May to June 2020. | N/A | | |
| Silveira  (2021)[47] | Brazil | "Evaluate extensive vaccination coverage data for invasive meningococcal disease, tuberculosis, DTP and pneumonia during the pandemic period as these vaccines are widely used in children's immunization programmes in Brazil and discuss the possible effects of the COVID-19 pandemic on public health in Brazil" | repeated cross-sectional (modelled data) | 1 January 2020 to 26 December 2020 | 1 January 2015 to 31 December 2019 | Vaccination coverage for DTaP and BCG dropped by approximately 20% in all regions. The decrease for PCV and meningococcal C conjugate vaccine (MCC) was approximately 10% in all regions. An assessment of different vaccine administration patterns found substantial fluctuation in vaccination drives (between 10% and 20%) across the country during the pandemic period. | DTap ~-20% (coverage)  BCG ~-20 (coverage)  PCV ~-10% (coverage)  MCC ~-10% (coverage) | | |
| Silveira  (2021)[46] | Brazil | "Estimate how many children missed immunisations since February 2020, when the first COVID-19 case was reported in Brazil" | (comparative) repeated cross-sectional | January 2020 to June 2020 | 2017-2019 | Coverage levels were considerably lower in 2020 compared to previous years except for the Pentavalent vaccine which only had a reduction of 7.2 percentage points. In 2020 coverage rates for BCG and Hepatitis B were stable over six months between 63% to 72% of the 2017 and 2019 values. For Pentavalent vaccine, polio and MMR, coverage ratios were substantially lower in March and April compared to earlier months. In June these levels returned to almost the same as previous years, particularly for the Pentavalent vaccine. Nineteen percent of children missed vaccines as reported in the questionnaire. Twenty-one percent of children missed vaccines as reported on the vaccine card. Infants were less likely to miss vaccinations than one-year olds. Results for two-year-olds were inconsistent, with low frequency according to the vaccine card. Children from poor families were more likely to miss vaccines. | Pentavalent -7.9 percentage points (coverage) | | |
| Sinuraya  (2022)[26] | Indonesia | "Investigate parents' knowledge, attitude, and practice on childhood immunization during the COVID-19 pandemic in Indonesia" | cross-sectional | June 2020 to February 2021 | N/A | Approximately 80% of parents had good knowledge of childhood vaccination. This was found to be influenced by level of education, occupation, level of knowledge and a positive attitude towards vaccination. There was a significant association between respondents' educational background and occupation with knowledge of childhood vaccination. Employed parents achieved better scores compared to unemployed parents. Side effects of vaccinations were poorly understood by respondents. | N/A | | |
| Torres  (2021)[56] | Argentina | "Assess the impact of the SARS-CoV-2 pandemic on the administration of the Pentavalent and the MMR vaccines to children younger than 2 years at the vaccination centre of children's hospital in Buenos Aires" | (comparative) repeated cross-sectional | January to May 2020 | January to May 2019 | From January to May 2019 7,263 vaccines were administered to children under 2 years. For the same period in 2020, 5,407 vaccines were administered to children under 2 years. During the isolation period in March 2020 the total number of vaccines dropped by 64.2%. The Pentavalent vaccine dropped by 74.9% and MMR by 55.1%. People living outside of Buenos Aires were more affected with a 71.1% reduction than those in Buenos Aires which experienced a 24.7% reduction. | **Isolation period**  Overall -64.2% (doses)  Pentavalent -74.9% (doses)  MMR -55.1% (doses) | | |
| Tegegne  (2021)[25] | Ethiopia | "Assess the challenge and status of immunization during COVID-19 and associated factors among children aged 10-23 months" | Mixed methods - cross sectional/qualitative (detailed interviews) | September 2, 2020, to October 21, 2020 | N/A | Overall, 62.2% of respondents with children aged 10 to 23 months reported that the child had not completed their immunisation schedule. Respondents reported that: 85.4% of their children had received both BCG and OPV; 83.75% had received PCV1 and Pentavalent1; 83.2% had received the rotavirus vaccine; and 61.4% had received MCV. Factors associated with an incomplete immunisation schedule were waiting more than 30 minutes at the health facility, a woman who delivered at home, illiterate mother, fear of contracting COVID-19 from a healthcare worker, women who thought COVID-19 care is given with other health services. | N/A | | |
| Wambua  (2022)[31] | Kenya | "Assess the indirect impact of COVID-19 on utilisation of immunisation and outpatient services in Kenya" | (comparative) repeated cross-sectional | January 2018 to March 2021 | N/A | Overall immunisation services remained unaffected. In March 2020 there were observed spikes in the administration of MCV. There was a significant increase in MCV in March 2020. Slight decrease in December 2020 may be attributed to the healthcare worker strike. | N/A | | |
| Wang  (2022)[70] | China | "Examine childhood vaccination delay, explore the association between vaccination delay and parental vaccine hesitancy, and assess childhood vaccination delays during the coronavirus disease pandemic in China" | repeated cross-sectional | September 20 and 20 October 2020 | N/A | Coverage for BCG, Hepatitis B, polio, DTP, MCV, JEV1 and Hepatitis A was more than 95% at 24 months. For children vaccinated in a timely fashion, Hepatitis B first dose was the highest at 95% with BCG the lowest at 44.6%. The delayed vaccination rate was significantly higher in 2020 compared to previous years. The highest delays were in February and March 2020 with 55.44% and 53.45% respectively. Vaccination delay was negatively associated with convenient access to vaccination clinics and immunisation service satisfaction. | N/A | | |
| Wang  (2022)[27] | China | "Assess changes in trends of parental attitudes toward routine childhood vaccines and COVID-19 vaccinations across different time periods in China" | cross-sectional | September to October 2020, February to March 2021, and May to June 2021 | N/A | Parents showed hesitancy toward childhood vaccination using the vaccination hesitancy scale across the three waves of surveys with 7.8% 15.1% and 5.5% respectively. Sex and self-reported health status were associated with parental vaccine hesitancy. Men were more likely to show hesitancy. Those who reported good health were less likely to be hesitant. The data showed a sudden increase in hesitancy between February and March 2021 prior to the introduction of the COVID-19 vaccination policy. | N/A | | |
| Wanyana  (2021)[35] | Rwanda | "Assess the change in the utilization of maternal and child health (MCH) services during the COVID-19 outbreak" | (comparative) repeated cross-sectional | March and April 2020 | March and April 2019 | There was a significant decrease for BCG, OPV1-2, Pentavalent1-2, PCV1-2 and Rota1-2. MR1 increased in Southern Province. These variations may be due to the continuation of community-based interventions in the South and the recent delivery and postnatal care awareness campaigns in Kigali. |  | | |

**References (continued from References list in main article)**

89. Miretu DG, Asfaw ZA, Addis SG. Impact of COVID-19 pandemic on vaccination coverage among children aged 15 to 23 months at Dessie town, Northeast Ethiopia, 2020. Human Vaccines and Immunotherapeutics. 2021;17(8):2427-36. doi: <http://dx.doi.org/10.1080/21645515.2021.1883387>.

90. Patel K, Nayak B, Rana S, Krishnan P, Tandale BV, Basak S, et al. Enablers and barriers towards ensuring routine immunization services during the COVID-19 pandemic: findings from a qualitative study across five different states in India. Transactions of the Royal Society of Tropical Medicine and Hygiene. 2022. doi: <https://dx.doi.org/10.1093/trstmh/trac011>.

1. [↑](#footnote-ref-1)
2. [↑](#footnote-ref-2)
