# Supplementary material for: Impact of COVID-19 on routine childhood immunisations in low- and middle-income countries: A scoping review
Source: PLOS Glob Public Health. 2023 Aug 23;3(8):e0002268. doi: 10.1371/journal.pgph.0002268 (PMC10446229; doi:10.1371/journal.pgph.0002268)
Supplement: S2 Table — (DOCX) [file pgph.0002268.s003.docx]

**Supplementary 2: search strategy**

| Concept A | Coronavirus |  |
| --- | --- | --- |
| MEDLINE | Subject headings (MeSH) | COVID-19 |
| MEDLINE, EMBASE, Global Health | Text words | ((wuhan and (coronavirus or corona virus)) or (coronavirus* and ("19" or "2019" or "2020" or "2021" or "2022"))).tw,kf,hw.  (2019 nCov or nCov 2019 or ncov19 or ncov 19 or novel coronavirus* or novel corona virus* or Severe Acute Respiratory Syndrome Coronavirus 2 or coronavirus disease 2019 or corona-virus disease 2019 or new coronavirus* or new corona-virus* or SARS-Coronavirus-2 or SARS-Coronavirus2 or SARS-Corona-Virus-2 or SARS-corona-virus2 or SARS-like coronavirus* or (2019-novel CoV or SARS-COV-2 or SARS-COV2 or sarscov2 or sarscov-2 or coronavirus-19 or covid19 or covid-19)).tw,kf,hw.  ((novel or new or nouveau or pandemic*) adj2 (CoV or covid or ncov or coronavirus or corona-virus)).tw,kf,hw. |
| Concept B | Routine immunisation |  |
| MEDLINE | Subject headings (MeSH) | bcg vaccination/ or influenza vaccination/ or measles vaccination/ |
| MEDLINE, EMBASE, Global Health | Text words | (((hepatitis B or hepatitis b virus or hep B or hepB) adj vaccin*) or ("h b vax" or "hb vax" or Fendrix or hepaccine b or hepagene or hepatavax or hepavaxx B or heplisav or heprecomb or heptavax b or hepuman or hevac b or supervax or tgp 943 or theradigm)).mp.  (((dtwp or "di te per" or diphtheria pertussis tetanus or diphtheria tetanus pertussis or pertussis diphtheria tetanus or diphtheria pertussis triple or diphtheria tetanus acellular pertussis or diphtheria tetanus whooping cough or DTAP or diteper anatoxal berna or DPT or DTP or tetanus diphtheria pertussis or triplo) adj (vaccin* or trivaccin*)) or ((dtwp or "di te per" or diphtheria pertussis tetanus or diphtheria tetanus pertussis or pertussis diphtheria tetanus or diphtheria pertussis triple or diphtheria tetanus acellular pertussis or diphtheria tetanus whooping cough or DTAP or diteper anatoxal berna or DPT or DTP or tetanus diphtheria pertussis or triplo) adj booster*)).mp.  (Kinrix or Pediarix or Pentacel or Quadracel or Vaxelis or acel immune or acel imune or acelluvax dpt or acelluvax DTP or adacel or "dt coq" or "anatoxal di te per berna" or booster tdap or boostertdap or boostrix or certiva or covaxis or "d t coq" or daptacel or "dif per tet all" or "diphtheria plus pertussis plus tetanus" or diteki booster* or ditekibooster* or dtap booster* or dtapbooster* or infanrix or neodiftepertus or pdt vax or pertugen or tdap booster* or tdap immun or tdapbooster* or tri immunol or triacelluvax or triaxis or tribaccin* or tripacel or tripedia or tripvac or tritanrix or trivax).mp.  (oral polio or oral poliovirus or trivalent OPV or Orimune or TOPV or Live Oral Trivalent or opol or opvero or oral poliomyelitis or oral virelon or orimune or polio sabin or sabin polio or polioral or poliosabin or sabin oral or ((polio or sabin or poliovirus or poliomyelitis or poliomyelitis virus or salk) adj vaccin*) or (inactivated polio* or antipolio* or Adacel Polio or Boostrix or buccapol berna or imovax polio or ipol or ipv virelon or polio salk sero or polio kovax or poliovaccin* or tri polio or z 5181 or z 5285 or z5181 or z5285) or poliovirus type 1 plus poliovirus type 3 oral live attenuated).mp.  (((Hib or h?emophilus or h?emophilus influenza? or b type influenza or h?emophilus influenza? b or prohibit) adj vaccin*) or (H?emophilus influenza? type b or h?emophilus b conjugate or ActHIB or act hib or hbpv or hib ompc or hib vax or hiberix or hibest or hibtiter or omnihib or pedvax hib or pedvaxhib or vaxem hib)).mp.  (((rotavirus* or rota virus or wc3) adj vaccin*) or (RotaTeq or Rotarix or rix 4414 or rix4414 or rotamune or rotarix or rotashield or rotateq)).mp.  (((pneumoco?cal polysa?charid* or pneumoco?cal or pnuimune or pneumoco?cus or pneumoco?cal conjugate* or pneumoco?cal 10 valent conjugat* or pneumoco?cal 13 valent conjugat* or pneumoco?cal 14 valent conjugat* or pneumoco?cal 23 valent conjugat* or pneumoco?cal 7 valent conjugat* or pneumoco?cal polysa?charid* conjugat* or polysa?charid* conjugat* or pneumoco?cal sa?charid* conjugat* or pneumoco?cus polysa?charid* or streptoco?cus pneumonia?) adj vaccin*) or (pneumovax or PCV15 or Vaxneuvance or PCV20 or Prevnar 20 or PPSV23 or Pneumovax23 or moniarix or "pcv 13" or pcv13 or "phid cv" or pneu immune or pneumo 23 or "13 valent adsorbed pneumococcal" or pneumopur or "pnu immune" or "pnu imune" or prevenar or prevenar13 or prevnar or streptopur or streptorix or synflorix)).mp.  (((mmr or measles mumps rubella or mumps measles rubella or "m r" or measles rubella mumps or "measles mumps and rubella virus" or mumps rubella measles or rubella measles mumps) adj vaccin*) or (Priorix tetra or ProQuad or immravax or "m m rvaxpro" or "m r ii" or "m r vax" or "m m rvaxpro" or "m r ii" or "mm rvaxpro" or "mmr ii" or "mmr vax" or mmr vax pro or mmrvaxpro or morupar or mumeru vax or pluserix or priorix or "r o r vax" or tri kovax or trimovax or triviraten or trivirix or virivac)).mp.  (((BGC or BCG or Bacill* Calmette Guerin or calmette guerin bacill* or calmette* or "b c g" or bcg cell wall or Calmette Guerin bacill* or tubercle bacill* or tuberculos?s) adj vaccin*) or (Onco BCG or TheraCys BCG or aeras 402 or aeras402 or antituberculosis or BCG kultur or bcg test or BCG vaccin or BCG vacina or BCG vaksine or BCG 10 or calgevax or eurocrine L3 or immucyst or immun bcg pasteur or monovax or mva 85a or mva85a or mycobax or onco tice or oncotice or pacis or pastimmun or theracys intravesical or tice bcg or ticebcg or vpm 1002 or vpm1002)).mp.  ((vaccin* or immuni#ation) adj2 (program* or schedule* or timetable* or agenda*)).mp.  ((routine or standard or regular or customary or accustomed or normal or usual or ordinary or established or typical or common or commonplace or conventional or habitual) adj (vaccin* or immuni#ation)).mp.  ((child* or infant* or newborn* or neonat* or p?ediatric) adj (vaccination* or immuni#ation*)).mp. |
| Concept C | Low-and middle-income country |  |
| MEDLINE | Subject headings (MeSH) | Developing Countries/  exp africa/ or exp caribbean region/ or exp central america/ or latin america/ or exp south america/ or asia/ or exp asia, central/ or exp asia, southeastern/ or exp asia, western/ or exp indian ocean islands/ or pacific islands/ or exp melanesia/ or exp micronesia/ or exp west indies/ |
| MEDLINE, EMBASE, Global Health | Text words | (Afghan* or Albania* or Algeria* or Angola* or Argentin* or Armenia* or Azerbaijan* or Bangladesh* or Belarus* or Beliz* or Benin* or Bhutan* or Bolivia* or Bosnia* or Herzegovin* or Botswan* or Brazil* or Bulgaria* or Burkina* or Burundi* or Cabo Verde* or Cape Verde* or Cambodia* or Cameroon* or Central Africa* or Chad* or China or Chinese or Colombia* or Comor* or Congo* or Costa Rica* or "Cote d'Ivoir*" or Ivory Coast or Cuba* or Djibouti* or Dominica* or Ecuador* or Egypt* or "El Salvador*" or Eritrea* or Ethiopia* or Fiji* or Gabon* or Gambia* or Georgia* or Ghana* or Grenad* or Guatemala* or Guinea* or Guyan* or Haiti* or Hondura* or Hungar* or India* or Indonesia* or Iran* or Iraq* or Jamaica* or Jordan* or Kazakhstan* or Kenya* or Kiribati* or Korea* or Kosov* or Kyrgyz* or Lao* or Leban* or Lesotho* or Liberia* or Libya* or Macedonia* or Madagascar* or Malawi* or Malay* or Maldiv* or Mali* or Marshall Island* or Mauritania* or Mauriti* or Mexic* or Micronesia* or Moldova* or Mongolia* or Montenegr* or Morocc* or Mozambi* or Myanma* or Burmese or Namibia* or Nepal* or Nicaragua* or Niger* or Nigeria* or Pakistan* or Panama* or Papua New Guinea* or Paraguay* or Peru* or Philippines or Filipino or Romania* or Rwanda* or Samoa* or Sao Tome* or Senegal* or Serbia* or Seychell* or Sierra Leon* or Solomon Island* or Somalia* or South Africa* or Sudan* or Sri Lanka* or "St Lucia*" or "St Vincent*" or Grenadine* or Surinam* or Swazi* or Syria* or Tajikistan* or Tanzania* or Thai* or Timor* or Togo* or Tonga* or Tunisia* or Turk* or Turkmenistan* or Tuvalu* or Uganda* or Ukrain* or Uzbekistan* or Vanuatu* or Venezuela* or Vietnam* or West Bank or Gaza or Yemen* or Zambia* or Zimbabwe*).mp.  (africa* or asia* or caribbean or central america* or latin america* or south america* or melanesia* or micronesia* or polynesia*).mp.  (resource-limit* or resource-poor or low-resource* or limited-resource* or resource-constrain* or constrain*-resource* or under-resource* or poor*-resource* or resource-scarce* or scarce*-resource* or low-income or middle-income or lowincome or middleincome or (low adj3 middle-income) or ((developing or underdeveloped or under-developed or emerging or less-developed or least-developed or less-economically developed or least-economically developed or less-affluent or least-affluent or deprived or poor) adj (country or countries or nation or nations or region or regions or economy or economies)) or ((developing or underdeveloped or under-developed or less-developed or least-developed) adj (population* or world))).mp.  (third-world* or thirdworld* or 3rd-world* or lmic or lmics or lami countr* or lalmi countr* or transitional countr* or (low* adj (gdp or gnp or gross domestic or gross national)) or ((underserved or under served) adj (countr* or nation? or population*))).mp. |
